# Development and validation of a predictive model for postoperative functional recovery in patients with spontaneous intracerebral hemorrhage

**DOI:** 10.3389/fsurg.2025.1589876

**Published:** 2025-10-17

**Authors:** Ziming Jiang, Ruijuan Zhang, Danfeng Weng, Yuhang Lv, Liang Dong

**Affiliations:** Department of Critical Care Medicine, Taizhou Central Hospital (Taizhou University Hospital), Taizhou, Zhejiang, China

**Keywords:** spontaneous intracerebral hemorrhage, predictive model, Boruta, logistic regression, nomogram

## Abstract

**Background:**

This study aimed to develop and validate a prognostic nomogram for predicting 3-month functional recovery in patients undergoing surgery for spontaneous intracerebral hemorrhage (ICH).

**Methods:**

A retrospective cohort of 289 patients diagnosed with spontaneous intracerebral hemorrhage (ICH) underwent surgical management at the Intensive Care Unit of Taizhou Central Hospital between January 2021 and December 2024 was enrolled. Patients were randomly allocated into a training set (*n* = 203, 70%) and validation set (*n* = 86, 30%). A prognostic nomogram integrating imaging characteristics and clinical parameters was developed to predict 90-day functional recovery (modified Rankin Scale ≤2). Feature selection employed the Boruta algorithm, followed by multivariable logistic regression. Model discrimination was quantified by area under the ROC curve (AUC), while calibration curve was performed to evaluate model performance. Clinical utility was evaluated through decision curve analysis (DCA).

**Results:**

The multivariable model retained six significant predictors: midline shift (OR:2.09, 95%CI: 1.56–2.79), hematoma volume (OR:1.10, 95%CI: 1.05–1.15), age (OR:1.03, 95%CI: 1.01–1.05), mean arterial pressure (OR:0.93, 95%CI: 0.89–0.98), body mass index (OR:0.78, 95%CI: 0.66–0.92), and Glasgow Coma Scale (GCS) score (OR:0.92, 95%CI: 0.79–1.06). Discriminative performance was robust, with area under the receiver operating characteristic curve (AUC) of 0.90 (95% CI: 0.85–0.96) in the training set and 0.83 (95% CI: 0.73–0.93) in the validation set. Calibration plots demonstrated excellent agreement between predicted and observed probabilities. DCA confirmed the clinical value of the model and its good impact on actual decision-making.

**Conclusion:**

This study developed and validated a pragmatic prognostic nomogram for spontaneous ICH patients undergoing surgical intervention, integrating six clinically actionable predictors: midline shift, hematoma volume, age, MAP, BMI, and GCS. The model demonstrated robust discriminative capacity, calibration and clinical applicability, which provides evidence-based support for the formulation of individualized rehabilitation programs and the optimization of medical resources.

## Introduction

Spontaneous intracerebral hemorrhage (ICH), defined as acute extravasation of blood into the brain parenchyma secondary to cerebrovascular rupture, is recognized as the most lethal form of acute stroke (1), carrying an early mortality rate of 30%–40% (2). In the United States, ICH accounts for approximately 10% of the estimated 795,000 annual stroke cases (3). Despite significant advancements in minimally invasive surgical techniques in recent years, postoperative functional recovery in patients demonstrates marked heterogeneity. Notably, no clinical outcome improvement was observed in preoperatively comatose patients undergoing surgical intervention (4). This prognostic uncertainty poses substantial challenges for clinical decision-making, rehabilitation resource allocation, and physician-patient communication. Current prognostic scoring systems (e.g., ICH Score, FUNC Score) incorporating parameters such as age, hematoma volume, and Glasgow Coma Scale (GCS) (5, 6), demonstrate significant limitations when applied to postoperative functional recovery prediction. Most existing models were developed using Western population datasets and have demonstrated suboptimal performance during external validation in Asian cohorts (7).

Prediction models are designed to forecast future outcomes based on a set of baseline predictors, thereby facilitating medical decision-making and improving patient prognostication (8), These models are increasingly employed in contemporary practice to assess and predict clinical endpoints. The publication of transparent reporting guidelines for prediction model studies [TRIPOD (Transparent Reporting of a Multivariable Prediction Model for Individual Prognosis or Diagnosis) Statement] (9), has established a standardized framework for developing prediction models with enhanced methodological rigor and clinical utility.

The primary objective of this retrospective study was to develop a predictive model for postoperative functional recovery in patients with spontaneous intracerebral hemorrhage (ICH). We sought to identify critical risk factors potentially influencing clinical outcomes. Utilizing clinical characteristics of ICH patients as predictors, a prognostic nomogram was constructed to facilitate individualized outcome assessment.

## Methods

### Data source

Patient data were retrospectively extracted from the electronic medical records of Taizhou Central Hospital. This study was approved by the Institutional Review Board (IRB) of Taizhou Central Hospital (Approval No.: 2025l-03-054). Informed consent was waived by the IRB due to the retrospective nature of the study.

We included consecutive patients meeting the following criteria:

1. Diagnosis: Spontaneous intracerebral hemorrhage (ICH) confirmed by non-contrast CT or MRI within 24 h of symptom onset.
2. Intervention: Underwent surgical management (hematoma evacuation or decompressive craniectomy) within 24 h of symptom onset.
3. Timeframe: Admitted between January 2021 and December 2024.

Exclusion criteria were:

1. Age <18 years
2. Delayed diagnosis (>24 h from symptom onset to imaging)
3. Secondary hemorrhage due to trauma, tumor
4. Palliative care-only approach per family request
5. Missing data exceeding 20% of key variables
6. Active malignancy or severe hepatic/renal insufficiency

**Figure 1:**
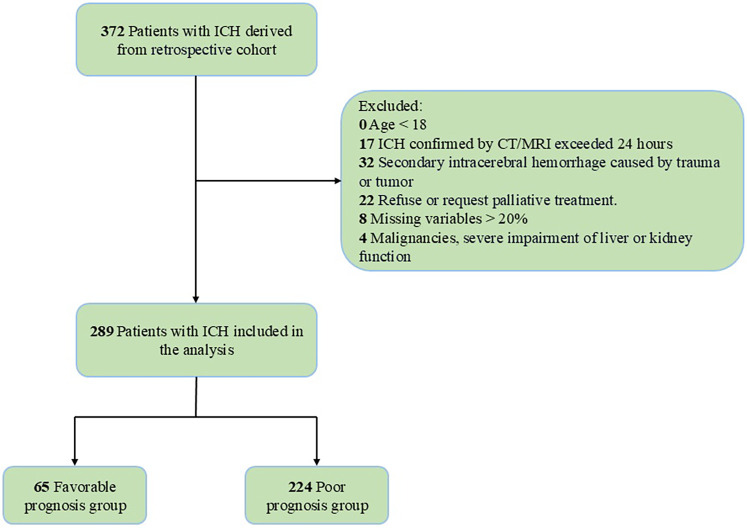
Flowchart of the study.

The study flowchart detailing enrollment is provided in Figure 1.

### Data collection

1. Demographic Data: Age, sex, BMI, Smoking, alcohol consumption
2. Medical history: hypertension, diabetes, anticoagulant use
3. Neuroimaging Assessment: Hemorrhage location (supratentorial yes/no), Hematoma volume calculated via ABC/2 formula (10), Midline shift measurement (the greatest degree of shift, millimeters) on axial CT
4. Physiological Parameters: Glasgow Coma Scale (GCS), temperature, SBP, DBP, MAP, heart rate, respiratory rate, SpO2
5. Laboratory Profiling: pH, PaO2, PaCO2, white blood cell, hemoglobin, platelet, potassium, sodium, blood urea nitrogen, creatinine, international normalized ratio, prothrombin time

### Outcome assessment

Functional outcomes were evaluated using the modified Rankin Scale (mRS) through structured telephone interviews at 3-month follow-up. Outcomes were dichotomized as favorable (mRS score ≤2) or poor (mRS score >2).

### Statistical analysis

**Figure 2:**
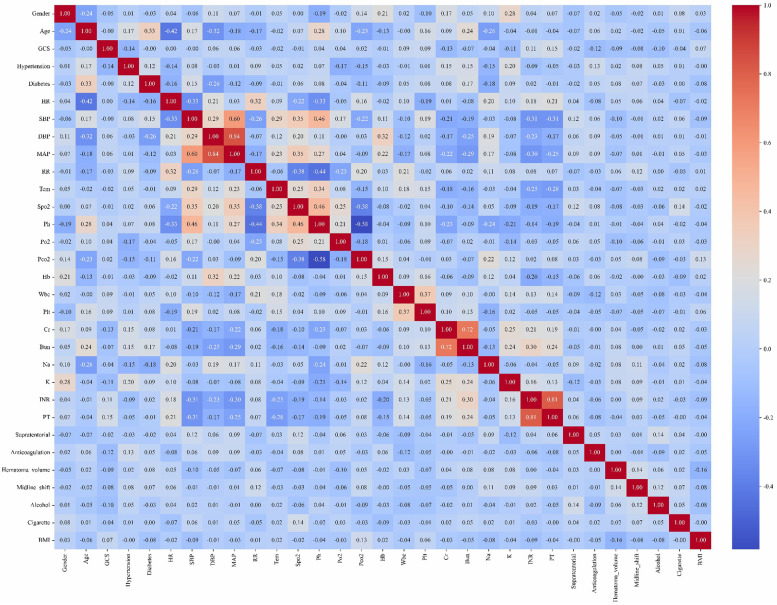
Correlation heatmap of variables.

Given the retrospective design of this study, *a priori* sample size calculation was not performed. Continuous variables were expressed as mean ± standard deviation (SD) when normally distributed (assessed by Shapiro–Wilk test), or median with interquartile range (IQR, 25th–75th percentiles) for skewed distributions. Categorical variables were reported as percentages (counts/total counts). For continuous variables, we perform a *t*-test or a Mann–Whitney *U*-test to compare between groups. For categorical variables, we use the chi-square test or Fisher's exact test. Variables with >20% missing data were excluded from multivariable analysis. For variables with ≤20% missingness, multiple imputation was implemented via the MICE package (Multivariate Imputation by Chained Equations) (11). Collinearity among predictors was systematically evaluated visualized in a correlation heatmap (Figure 2). All analyses were performed using R Statistical Software (v4.4.2) and Python (v3.9).

## Results

### Baseline characteristics

This study ultimately included 289 patients with spontaneous intracerebral hemorrhage (ICH), among whom 65 patients achieved favorable outcomes (mRS ≤ 2), and 224 patients had poor outcomes (mRS > 2). Baseline characteristics such as alcohol consumption history (*p* = 0.069), hypertension (*p* = 0.051), diabetes (*p* = 0.091), supratentorial hemorrhage location (*p* = 0.57), and anticoagulant use (*p* = 0.69) showed no significant differences between the groups. Physiological parameters, including SBP (*p* = 0.723), heart rate (*p* = 0.38), respiratory rate (*p* = 0.086), pH (*p* = 0.765), PaCO₂ (*p* = 0.297), PaO₂ (*p* = 0.329), and laboratory markers such as white blood cell count (*p* = 0.219), hemoglobin (*p* = 0.235), and serum sodium (*p* = 0.065), also demonstrated no significant intergroup differences. However, the poor prognosis group exhibited statistically significant differences (*p* < 0.05) in multiple critical predictors compared to the favorable prognosis group. The poor prognosis group was older, had a higher proportion of females, and showed elevated smoking history prevalence; larger hematoma volume and greater midline shift; lower BMI and GCS scores, higher body temperature, and reduced mean arterial pressure, diastolic blood pressure, and oxygen saturation; lower platelet counts, elevated potassium, blood urea nitrogen, creatinine, international normalized ratio, and prothrombin time (Table 1).

**Table 1:** Baseline characteristics.

Characteristics	Favorable prognosis group (*N* = 65)	Poor prognosis group (*N* = 224)	*P*-value
Age (years)	51.90 [31.66, 79.56]	69.80 [51.33, 83.27]	0.002
Male (%)	50 (76.92)	138 (61.61)	0.023
BMI (kg/m^2^)	27.18 ± 3.36	25.27 ± 2.44	<0.001
Alcohol (%)	34 (52.31)	145 (64.73)	0.069
Cigarette (%)	33 (50.77)	160 (71.43)	0.002
Hypertension (%)	55 (84.62)	163 (72.77)	0.051
Diabetes (%)	55 (84.62)	167 (74.55)	0.091
Supratentorial (%)	54 (83.08)	179 (79.91)	0.570
Anticoagulation (%)	13 (20.00)	50 (22.32)	0.690
Hematoma volume (mL)	36.54 [33.45, 41.82]	45.68 [39.27, 52.08]	<0.001
Midline shift (mm)	3.29 [1.97, 5.35]	5.09 [4.25, 6.35]	<0.001
GCS	11.00 [7.00, 13.00]	8.50 [7.00, 11.00]	0.011
Temperature (℃)	37.23 [36.86, 37.57]	36.92 [36.43, 37.58]	0.008
SBP (mmHg)	119.11 [113.03, 127.77]	121.07 [108.58, 131.69]	0.723
MAP (mmHg)	80.69 [75.83, 87.05]	78.67 [71.65, 84.53]	0.017
DBP (mmHg)	64.87 ± 9.65	60.94 ± 10.76	0.006
HR (/min)	82.21 [73.83, 96.29]	87.46 [74.76, 102.61]	0.380
RR (/min)	18.44 [16.50, 20.90]	19.07 [16.87, 22.50]	0.086
Spo2 (%)	99.12 [98.25, 99.82]	98.63 [96.84, 99.63]	0.013
Ph	7.38 [7.32, 7.44]	7.38 [7.30, 7.44]	0.765
PaCO₂ (mmHg)	41.00 [35.50, 45.00]	38.00 [34.00, 44.62]	0.297
PaO₂ (mmHg)	190.00 [144.50, 260.00]	187.00 [129.25, 262.62]	0.329
Wbc (*10^9^/L)	11.95 [9.05, 15.30]	12.86 [9.39, 16.08]	0.219
Hb (g/L)	11.36 ± 1.86	11.04 ± 2.18	0.235
Plt (*10^9^/L)	192.50 [161.50, 227.50]	169.50 [134.50, 227.50]	0.037
K (mmol/L)	4.00 [3.65, 4.40]	4.20 [3.80, 4.55]	0.044
Na (mmol/L)	140.00 [138.00, 142.00]	141.00 [138.00, 144.00]	0.065
Bun (mg/dL)	14.00 [11.50, 19.00]	18.50 [14.00, 26.00]	<0.001
Cr (mg/dL)	0.90 [0.75, 1.10]	1.10 [0.80, 1.45]	0.006
INR	1.20 [1.10, 1.35]	1.25 [1.15, 1.55]	0.002
PT (s)	12.85 [12.10, 14.45]	13.97 [12.60, 17.11]	0.003

BMI, body mass index; GCS, Glasgow Coma Scale; SBP, systolic blood pressure; MAP, mean arterial pressure; DBP, diastolic blood pressure; HR, heart rate; RR, respiratory rate; Hb, hemoglobin; Plt, platelet; INR, international normalized ratio; PT, prothrombin time.

### Feature selection

**Figure 3:**
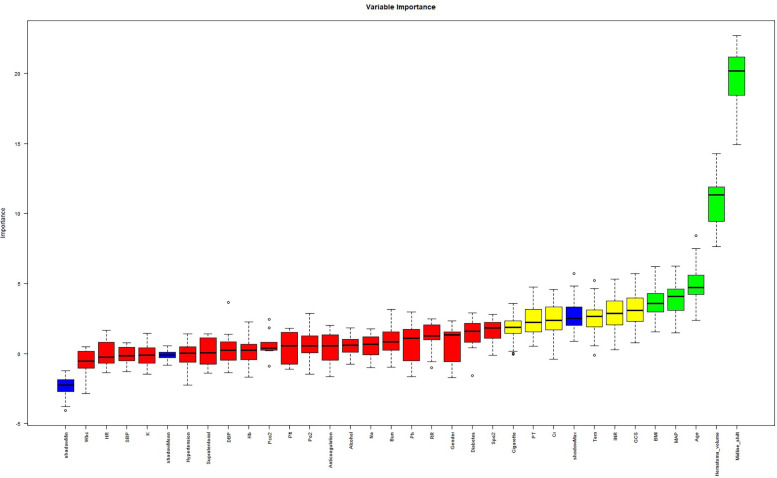
Boruta algorithm for feature importance analysis.

The cohort was randomly stratified into training (70%) and validation (30%) sets. Feature selection was performed in the training set using the Boruta algorithm, a robust wrapper method based on random forest classification. Unlike conventional feature selection approaches that optimize for model-specific performance, Boruta identifies features with intrinsic relevance to the outcome variable by iteratively comparing original attributes to shadow features (random permutations) (12). From 31 candidate predictors, Boruta selected five features with permutation importance: midline shift, hematoma volume, age, MAP, BMI (Figure 3). It is well established that the GCS is a classical tool for assessing the level of consciousness in patients with brain injury and is widely used for prognostic evaluation in neurological disorders such as spontaneous intracerebral hemorrhage (13–15). Numerous clinical studies have demonstrated a close association between GCS scores and adverse outcomes in patients with cerebral hemorrhage (16–18). In clinical practice, GCS also serves as a critical reference for physicians in evaluating the severity of a patient's condition and guiding clinical decision-making. The ultimate goal of predictive modeling is not only to achieve statistical significance but also to ensure clinical interpretability and applicability (19). Therefore, despite the lack of statistical significance between GCS and prognosis in our multivariate regression analysis (*p* = 0.25), we have included GCS in the final model, acknowledging its established clinical relevance. In the Supplementary Material, we further compared model performance between versions incorporating and excluding the GCS. The final multivariable logistic regression model included these six predictors.

### Nomogram

**Figure 4:**
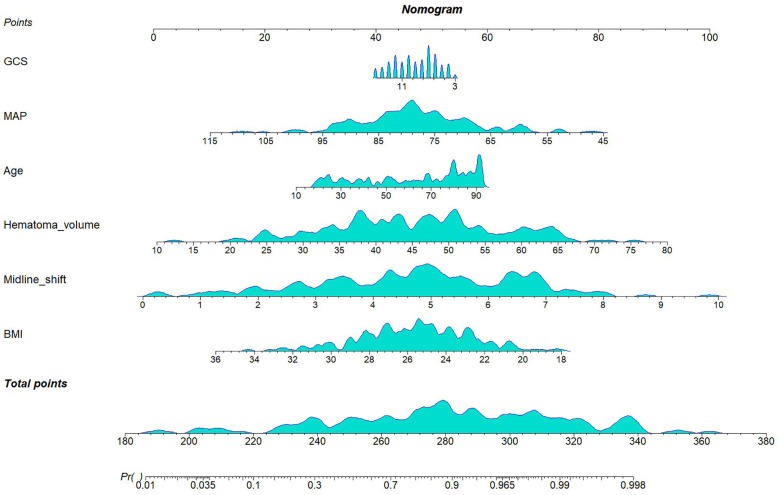
Nomogram for the final model.

In the training cohort (*n* = 202), the six selected predictors: midline shift, hematoma volume, age, MAP, BMI, and GCS, were incorporated into a multivariable logistic regression model. The final model demonstrated the following adjusted odds ratios (aORs) with 95% confidence intervals (Table 2): BMI (OR: 0.78; 95%CI 0.66–0.92), midline shift (OR: 2.09; 95%CI 1.56–2.79), hematoma volume (OR: 1.10; 95%CI 1.05–1.15), age (OR: 1.03; 95%CI 1.01–1.05), MAP (OR: 0.93; 95%CI 0.89–0.98), GCS (OR: 0.92; 95%CI 0.79–1.06). A clinical nomogram integrating these predictors was constructed, assigning weighted points proportional to each variable's β-coefficient (Figure 4).

**Table 2:** Multivariable logistic regression model.

Variables	Multivariable logistic model		
	OR	95%CI	*P*-value
BMI	0.78	0.66–0.92	0.003
Midline shift	2.09	1.56–2.79	<0.001
Hematoma volume	1.10	1.05–1.15	<0.001
Age	1.03	1.01–1.05	0.003
MAP	0.93	0.89–0.98	0.004
GCS	0.92	0.79–1.06	0.25

BMI, body mass index; MAP, mean arterial pressure; GCS, Glasgow Coma Scale; OR, odds ratio; CI, confidence interval.

## Model performance evaluation

### Discrimination

**Figure 5:**
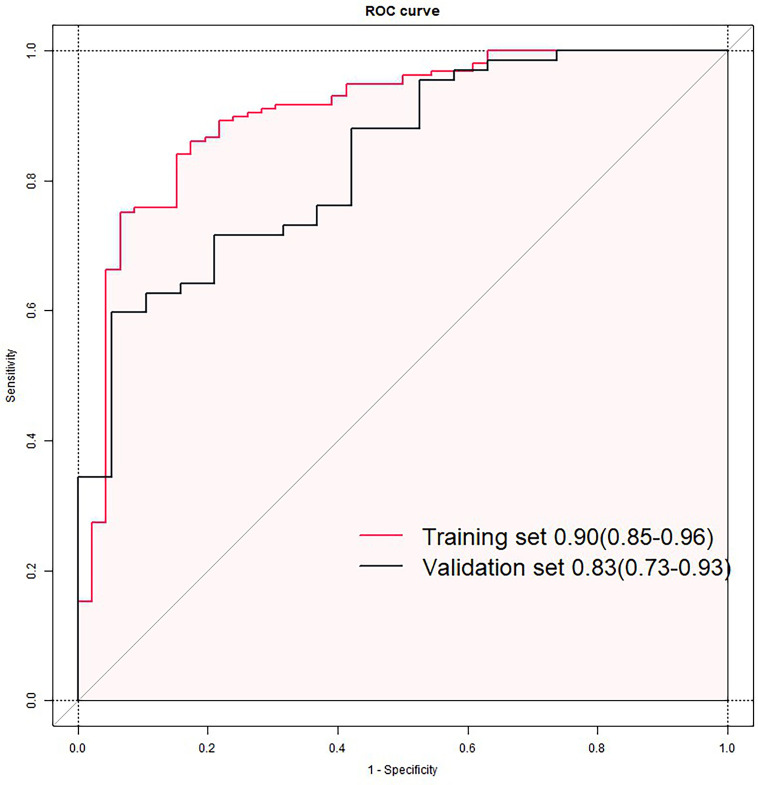
Receiver operating characteristic (ROC) curves of the nomogram model in training **(A)** and validation sets **(B)**.

The prognostic model derived from multivariable logistic regression was rigorously evaluated using receiver operating characteristic (ROC) curve analysis. In the training cohort, the model demonstrated exceptional discriminative ability with an area under the curve (AUC) of 0.90 (95% CI: 0.85–0.96). When applied to the validation set, the AUC remained clinically significant at 0.83 (95% CI: 0.73–0.93), showing a moderate yet meaningful predictive capacity despite expected performance attenuation (Figure 5).

### Calibration

**Figure 6:**
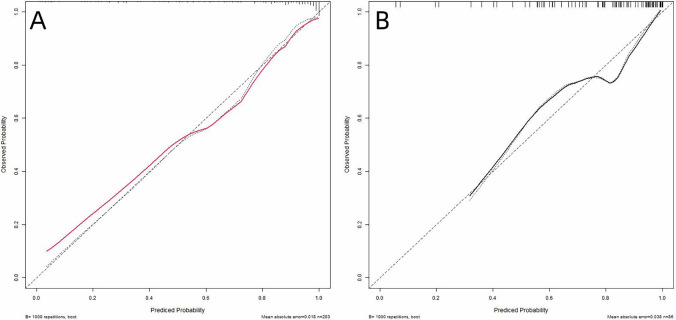
Calibration curves of the nomogram in training **(A)** and validation **(B)** sets. The x-axis represents the predicted probability of poor prognosis calculated by the nomogram. The *y*-axis indicates the observed probability of adverse outcomes. The 45-degree dashed line (y = x) corresponds to perfect prediction alignment.

Calibration performance was rigorously evaluated using calibration plots with 95% confidence intervals, comparing predicted probabilities of poor outcome against observed frequencies. Perfect calibration aligns with the 45-degree reference line (intercept = 0, slope = 1). Visual inspection revealed satisfactory agreement between predictions and observations in both sets (Figure 6).

### Decision curve analysis

**Figure 7:**
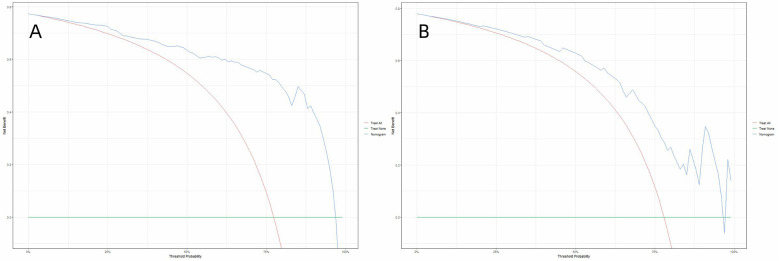
Decision curve analysis (DCA) of the nomogram in the training set **(A)** and the validation set **(B)** the decision curve analysis demonstrated that the nomogram provided superior net benefit compared to the “treat-all” and “treat-none” strategies across a threshold probability range of 4%–95% **(A)** and 5%–94% **(B)**.

To evaluate the model's clinical practicality, decision curve analysis (DCA) was conducted by quantifying the net benefit across varying threshold probabilities, the minimum predicted risk at which intervening is deemed justified. Unlike conventional metrics (e.g., AUC), DCA directly reflects clinical utility by weighing benefits (true positives) against harms (false positives) through the formula. By plotting clinical decision curves, we found that the model exhibited a higher net benefit within threshold probability ranges of 4%–95% and 5%–94% for the training and validation sets, respectively (Figure 7). Decision curve analysis demonstrates that the use of this nomogram provides a higher net benefit than treating all or no patients, across a range of clinically relevant threshold probabilities. For example, if a clinician wishes to intensify rehabilitation for patients with a predicted risk above 30%, the nomogram can identify appropriate candidates, thereby optimizing resource allocation and improving patient outcomes.

## Discussion

In this study, poor functional outcome was defined as mRS score >2, while favorable outcome was defined as mRS score ≤2. Our final model identified six robust predictors of postoperative functional recovery: midline shift, hematoma volume, age, MAP, BMI, and GCS score. Notably, this prognostic tool integrating both neuroimaging features (midline shift, hematoma volume) and MAP with traditional clinical variables in spontaneous ICH surgical cohorts. This provides clinicians with a valuable tool for prognostic assessment in such patients, enhancing the precision of postoperative outcome prediction.

According to the Monro-Kellie doctrine (20), the total volume of intracranial contents (brain tissue, blood, and cerebrospinal fluid) remains constant. An increase in hematoma volume or elevated intracranial pressure may lead to brain tissue displacement (such as midline shift) and impaired neurological function (e.g., decreased GCS score). Midline shift, hematoma volume, and age have been consistently identified as key prognostic determinants in patients with intracerebral hemorrhage (5, 21, 22), primarily mediated through the mass effect resulting from the combined impact of parenchymal destruction and mechanical compression by the hematoma (4, 23). Yang et al. (24) demonstrated that a midline shift >4 mm represents the optimal cutoff value significantly associated with poor outcomes in ICH patients. Hematoma volumes exceeding 30 ml showed statistically significant correlation with adverse outcomes, while the combination of hematoma volume >60 ml and GCS score <8 predicted 30-day mortality rates exceeding 90% (5). Early-stage blood pressure control emerges as a critical therapeutic intervention to prevent hematoma expansion, with acute-phase antihypertensive therapy showing a modest but significant effect in reducing hematoma growth (25). Recent evidence (26) confirms that achieving and maintaining systolic blood pressure (SBP) within 120–130 mmHg during the initial 24-hour window demonstrates optimal safety and potential functional benefits. Given hypertension's status as a predominant etiological factor in ICH pathogenesis, our findings revealing the association between MAP and postoperative functional recovery. Notably, higher MAP levels demonstrated a significant protective correlation, aligning with conventional cerebral perfusion pressure (CPP) maintenance targets (27). Specifically, maintaining higher MAP was associated with in favorable outcomes. These results underscore the imperative for implementing dynamic hemodynamic monitoring protocols throughout the perioperative period. In our cohort, elevated body mass index (BMI) demonstrated a significant protective association with functional outcomes (OR 0.78; 95%CI 0.66–0.92). This finding aligns with the emerging concept of the “obesity paradox” in neurocritical care, where overweight status confers survival advantages in acute brain injury populations (28–30). The mechanisms underlying the obesity paradox remain incompletely elucidated, but are believed to involve multifactorial pathways including increased sympathetic nervous system activity, enhanced mitochondrial metabolic capacity, and elevated serum lipoproteins levels (31). Another plausible explanation for the protective effect of BMI observed in this study is that it may serve as a proxy for overall nutritional status. The increased metabolic reserves associated with fat or muscle mass in individuals with higher BMIs may contribute to their ability to withstand inflammatory events during the treatment period (32). Potential inconsistencies concerning the obesity paradox might stem from the higher incidence of complications associated with morbid obesity. For instance, the established link between obesity and insulin resistance (33), where diabetes can lead to poorer outcomes following ICH (34), warrants consideration. Further research, involving large-scale studies and relevant randomized controlled trials (RCTs), is necessary to elucidate the complex relationship between obesity and ICH prognosis. Previous studies (35) have identified INR as an independent risk factor for 3-month mortality in patients with spontaneous intracerebral hemorrhage, potentially due to its association with the use of oral anticoagulants such as warfarin. Additionally, early temperature changes within the first 24 h following intracerebral hemorrhage have been linked to poorer functional outcomes (36). In our cohort, however, when considered alongside other predictors, INR and body temperature did not demonstrate a strong independent association with postoperative functional recovery. This may be attributed to the limited distribution and variance of INR and temperature in our sample. For instance, most patients may have had INR values within the normal range, or temperature fluctuations may have been minimal. It is important to emphasize that the exclusion of these variables from our model does not diminish their clinical significance. Rather, it reflects the data-driven nature of the feature selection process in our specific cohort. Further studies involving larger and more diverse populations may help to clarify the prognostic value of these variables.

Despite its clinical utility, this study has limitations inherent to single-center retrospective designs, including potential selection bias and lack of *a priori* sample size estimation. The sample size was determined by the number of available cases. Future prospective studies with an adequate sample size are needed for further validation. External validation in diverse healthcare settings and integration of real-time biomarker dynamics could enhance predictive precision. Future implementation studies should evaluate the nomogram's impact on shared decision-making and resource allocation in neurocritical care units. The study did not compare the proposed model with existing clinical prediction models (such as the FUNC score). This limitation should be addressed in future prospective, multicenter studies through prospective collection of the necessary data.

## Conclusions

This study developed and validated a pragmatic prognostic nomogram for spontaneous ICH patients undergoing surgical intervention, integrating six clinically actionable predictors: midline shift, hematoma volume, age, MAP, BMI, and GCS. The model demonstrated robust discriminative capacity, calibration and clinical applicability, which provides evidence-based support for the formulation of individualized rehabilitation programs and the optimization of medical resources.

## Data Availability

The raw data supporting the conclusions of this article will be made available by the authors, without undue reservation.
