# Supplementary material for: Development and validation of a predictive model for postoperative functional recovery in patients with spontaneous intracerebral hemorrhage
Source: Front Surg. 2025 Oct 17;12:1589876. doi: 10.3389/fsurg.2025.1589876 (PMC12575222; doi:10.3389/fsurg.2025.1589876)
Supplement: Supplementary file 1 [file Datasheet1.pdf]

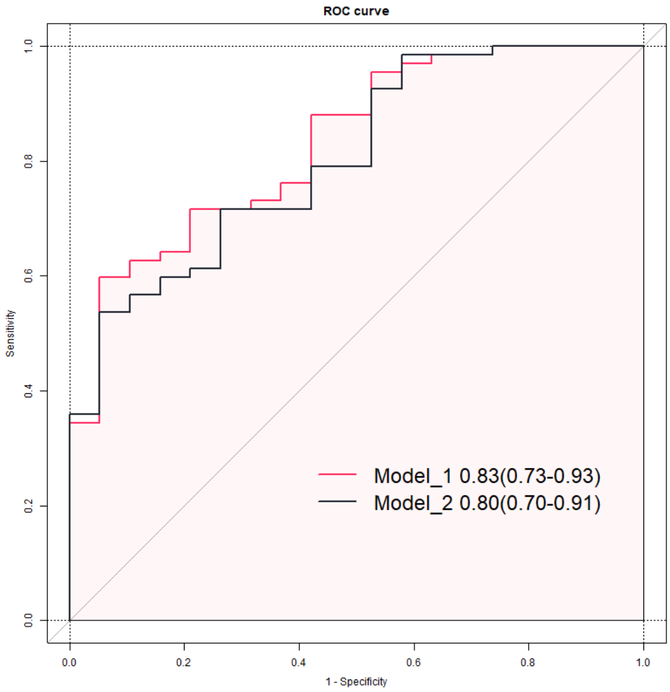

Fig.S1: Model 1: Include GCS score; Model 2: Exclude GCS score

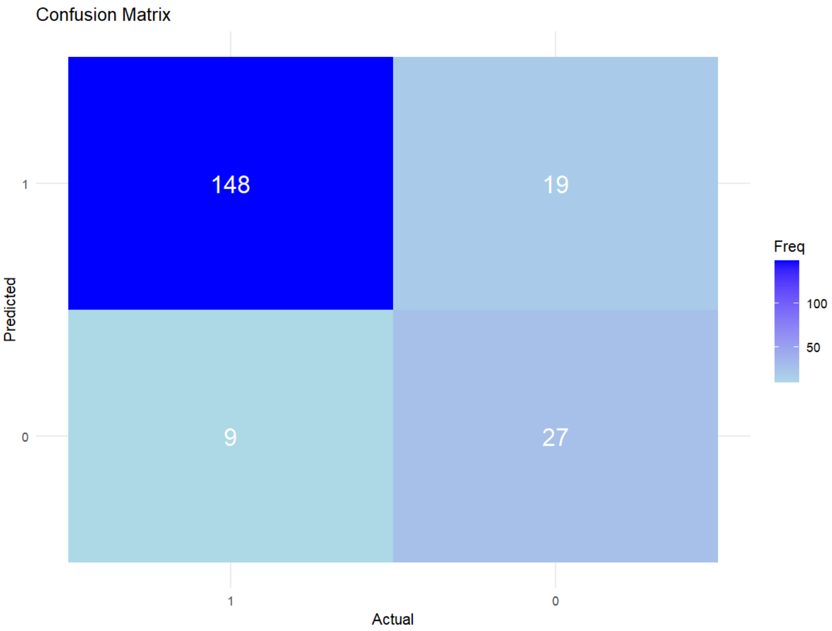

Fig.S2: Confusion Matrix (Training set)

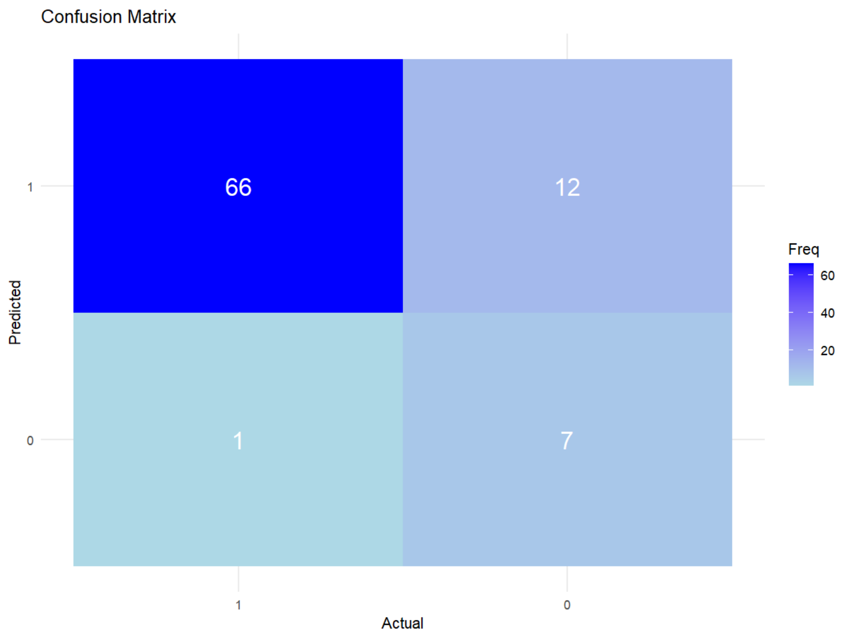

Fig.S3: Confusion Matrix (Validation set)

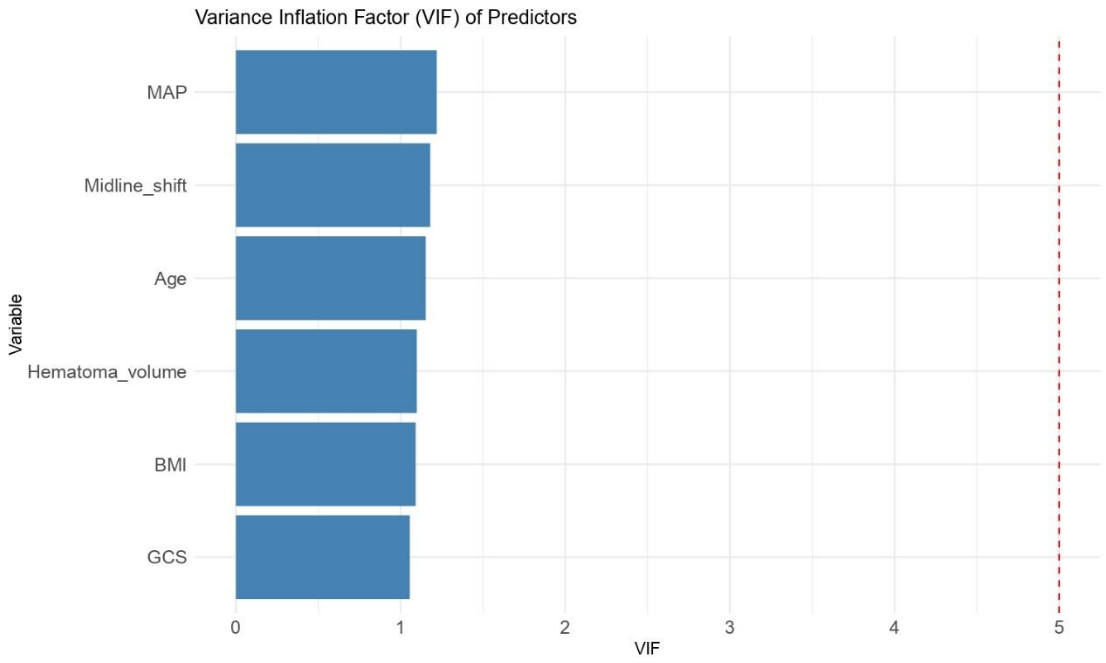

Fig.S4: Variance Inflation Factor(VIF) of Predictors

Table S1: The performance of different models in the validation set

	Accuracy	Precision	Recall	F1 Score	AUC
Model 1	0.87	0.88	0.97	0.92	0.77
Model 2	0.76	0.79	0.93	0.86	0.72
Model 3	0.81	0.82	0.97	0.89	0.74
Model 4	0.85	0.85	0.99	0.91	0.83

Model 1: BMI + Hematoma_volume + Age + MAP + GCS

Model 2: BMI + Midline_shift + Age + MAP + GCS

Model 3: BMI + Hematoma_volume + Age + Midline_shift + GCS

Model 4: BMI + Hematoma_volume + Age + Midline_shift + MAP + GCS
